# The Association of Coronary Fat Attenuation Index Quantified by Automated Software on Coronary Computed Tomography Angiography with Adverse Events in Patients with Less than Moderate Coronary Artery Stenosis

**DOI:** 10.3390/diagnostics13132136

**Published:** 2023-06-21

**Authors:** Wenzhao Zhang, Peiling Li, Xinyue Chen, Liyi He, Qiang Zhang, Jianqun Yu

**Affiliations:** 1 Department of Radiology, West China Hospital, Sichuan University, Chengdu 610041, China; 2 Department of Critical Care Medicine, Chengdu Shangjinnanfu Hospital, Chengdu 611730, China; 3 CT Collaboration, Siemens Healthineers, Chengdu 610041, China; 4 Department of Epidemiology and Biostatistics, West China School of Public Health and West China Fourth Hospital, Sichuan University, Chengdu 610041, China

**Keywords:** coronary heart disease, PCAT, machine learning, FAI, vascular inflammation

## Abstract

Objective: This study analyzed the relationship between the coronary FAI on CCTA and coronary adverse events in patients with moderate coronary artery disease based on machine learning. Methods: A total of 172 patients with coronary artery disease with moderate or lower coronary artery stenosis were included. According to whether the patients had coronary adverse events, the patients were divided into an adverse group and a non-adverse group. The coronary FAI of patients was quantified via machine learning, and significant differences between the two groups were analyzed via *t*-test. Results: The age difference between the two groups was statistically significant (*p* < 0.001). The group that had adverse reactions was older, and there was no statistically significant difference between the two groups in terms of sex and smoking status. There was no statistical significance in the blood biochemical indexes between the two groups (*p* > 0.05). There was a significant difference in the FAIs between the two groups (*p* < 0.05), with the FAI of the defective group being greater than that of the nonperforming group. Taking the age of patients as a covariate, an analysis of covariance showed that after excluding the influence of age, the FAIs between the two groups were still significantly different (*p* < 0.001).

## 1. Introduction

Coronary atherosclerotic heart disease is a type of heart disease that causes serious harm to human health, and it has ranked first in the composition of disease deaths among Chinese residents for some time [1]. Coronary artery wall atherosclerosis and its degree of stenosis are closely related to the prognosis of coronary heart disease. Coronary computed tomography angiography (CCTA) can more accurately judge the degree of coronary atherosclerotic plaques and lumen stenoses, so it plays an important role in the diagnosis and treatment of coronary heart disease by indirectly reflecting the degree of myocardial ischemia [2]. Currently, the observation of coronary arteries on CTA images is mostly limited to one-dimensional measurements of the long axis of the lesion or two-dimensional measurements of the lesion volume. Some information that is difficult to recognize by the human eye in the images has not been fully utilized, and its potential value in disease diagnosis and treatment cannot be fully realized [3,4]. The clinical diagnosis of coronary heart disease may not be consistent with the manifestations of CCTA [5], and there is currently no effective noninvasive detection method for distinguishing and diagnosing coronary artery stenosis from myocardial ischemia caused by stress [6].

Coronary adipose tissue (PCAT) is a part of epicardial fat that is mainly distributed around the outer membrane of the three main branches of the coronary artery. In recent years, studies have suggested that PCAT and coronary atherosclerosis may have an interactive relationship [7]. The objective is to capture early changes in coronary atherosclerosis by mapping the changes in perivascular fat attenuation using CCTA [8]. The fat attenuation index (FAI) around blood vessels is a novel quantitative value that has recently been proposed. It is defined as the weighted average attenuation in voxels (−190 to −30 Hounsfield units [HU]) of all adipose tissue within a radial distance from the outer blood vessel wall with equal diameter to each vessel. Its changes and role in coronary heart disease are receiving attention [9].

In the past, the measurement of pericoronal fat was mostly performed manually, which involved a time-consuming and tedious process. Moreover, previous studies primarily focused on measuring local volume and thickness in PCAT [10]. However, the quantification involved numerous processing steps, leading to concerns regarding the reproducibility of these measures. Consequently, the lack of consistent and reliable measurements has impeded research progress in this evidence-based medical domain [11]. To address these limitations, Siemens developed a software solution that incorporates machine learning algorithms which automate quantification of the Fat Attenuation Index (FAI). This innovative software offers a streamlined workflow with carefully pipelined process steps. Moreover, user interaction is facilitated at each stage to ensure accurate and reliable results, thereby mitigating the risk of obtaining flawed measurements. By incorporating machine learning and user-friendly features, Siemens’ software solution provides an enhanced and efficient approach to FAI quantification in medical imaging [12].

Moderate or more severe stenosis of the coronary artery often leads to adverse events in patients with coronary heart disease. However, during the follow-up process for coronary heart disease patients, the author found that some patients with moderate or lower stenosis of the coronary lumen still experienced coronary adverse events. Previous literature has not studied the causes of such patients’ occurrences. Therefore, this study used machine learning to quantify the coronary artery FAI (referred to as coronary artery FAI hereafter) in patients with moderate stenosis or lower (excluding moderate stenosis) in CCTA, and explored the relationship between the FAI and the occurrence of adverse events in coronary heart disease.

## 2. Materials and Methods

### 2.1. Research Subjects

The patient inclusion criteria were as follows: ① the patient underwent coronary artery CTA vascular examination at our hospital from January 2012 to December 2012 due to reasons such as “coronary heart disease” and “chest pain”; and ② these patients were followed up until September 2022. The degree of stenosis in the patient’s vascular lumen was below moderate (i.e., excluding moderate stenosis).

The patient exclusion criteria were the following: ① all-cause mortality patients other than noncardiac-related diseases; and ② patients whose images could not be analyzed with artificial intelligence software for postprocessing due to image quality (such as motion artifacts, respiratory artifacts, stent artifacts, etc.).

A total of 172 patients who met the inclusion and exclusion criteria were ultimately included in this study.

### 2.2. Basic Clinical Information of Patients

All of the patients recorded their age, sex, and smoking history during the CCTA examination, and fasting venous blood was collected from all patients who received the examination within one week of the CTA examination. Through the examination and analysis of venous blood, the corresponding results of blood sugar, total cholesterol, low-density cholesterol, high-density cholesterol, and triglycerides were obtained. At the same time, it was recorded whether these patients experienced coronary adverse events during the follow-up period, including coronary heart disease readmission, revascularization, myocardial infarction, patient death, etc.

### 2.3. Coronary Artery CTA Scanning Protocol

Using a 128-layer dual-source computer tomography (DSCT) scanner (Somatom Definition Flash; Siemens Medical Solutions, Forchheim, Germany) for image acquisition, retrospective ECG gating was used throughout the acquisition process. Before the examination, a detailed inquiry was made about the patient’s relevant medical history to confirm that the patient had a sinus rhythm, and that the heart rate was stable within the examination’s range of indications. When the patient had no contraindications and their resting heart rate was too fast or irregular, they could take β receptor blockers one hour before the examination to control their heart rate. The patients were asked about their height and weight, their BMI was calculated, and appropriate scanning parameters were selected. The patients were asked if they had a history of hypotension. If there was no hypotension and the heart rate was below 100 beats per minute, a small amount of nitroglycerin was sprayed under the patient’s tongue 3–5 min before the examination.

The specific parameters used were as follows: the scanning layer thickness was 0.6–0.75 mm; when the BMI value was less than 25, the tube voltage was 100 kV, and the tube current was 220 mAs; when the BMI value was greater than 25, the tube voltage was 120 kV, and the tube current was 300 mAs. The scanning range began at 1–2 cm below the bifurcation of the trachea and ended at the diaphragmatic surface of the heart, including all coronary arteries. The contrast agent injection method was intravenous injection of physiological saline ((6.0 mL/s, 20 mL) (test) + Ultravist 370; Bayer Pharma AG, Berlin, Germany) with contrast agent ((5.0 mL/s) + physiological saline (4.0 mL/s, 20 mL)). The dosage of contrast agent was 1–1.5 mL/kg, with a total volume of 50–90 mL. Delay scanning was performed using an automatic triggering method, with a scanning threshold of 100 HU and ROI in the ascending aorta.

For image postprocessing, the image (including the optimal diastolic and systolic images) was transmitted to the extended brilliancy workstation (EBW) for processing and reconstruction. The reconstruction parameters were as follows: layer thickness 0.75 mm, interval 0.5 mm, matrix 512 × 512.

### 2.4. CCTA Image Analysis

Three radiologists with >5 years of work experience were selected to analyze the patients’ CCTA images according to the Coronary Artery Disease Reporting and Data System criteria. Coronary artery stenoses were divided into six degrees: not visible: 0%; minimal: 1–24%; mild: 25–49%; moderate: 50–69%; severe: 70–99%; and occluded: 100% [13].

### 2.5. Measurement of the FAI Value of Fat around the Coronary Artery

Machine learning software (Siemens research prototype, CT Coronary Plaque Analysis, software version 5.0.2, Siemens-healthineers GmbH; Forchheim, Germany) was utilized to postprocess the CCTA images of all of the patients and obtain their FAI values [14]. To avoid differences in the length of the left coronary artery main stem and the influence of the aortic wall, the right coronary artery with a starting diameter of 10 mm and the left coronary artery main stem were excluded from this study. The FAIs of the LAD, LCX proximal 40 mm, and RCA proximal 10–50 mm were measured [15,16].

**Figure 1:**
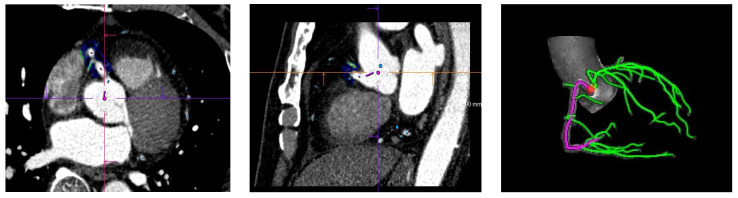
Coronary artery reconstruction: CTA data were imported into the Siemens research prototype FAI analysis software (CT Coronary Plaque Analysis, software version 5.0.2, Siemens-healthineers GmbH; Forchheim, Germany), which automatically performed 3D reconstruction of the LAD, LCX, and RCA.

**Figure 2:**
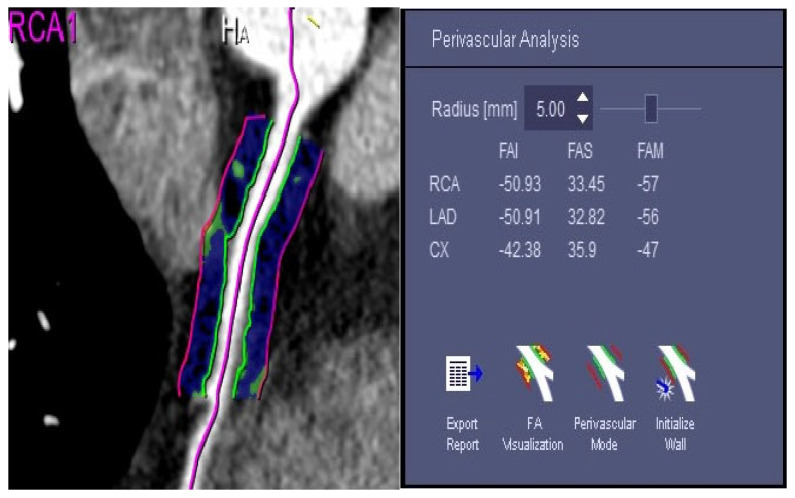
Measurement of FAI values: the target vascular segment was manually determined, and artificial intelligence software (CT Coronary Plaque Analysis, software version 5.0.2, Siemens-healthineers GmbH; Forchheim, Germany) was used to automatically outline the vascular wall (green line), and to automatically calculate the corresponding segment FAI value.

Specifically, the patient’s CCTA images were imported into machine learning software, and three-dimensional reconstruction of the left anterior descending branch (LAD), left circumflex branch (LCX), and right coronary artery (RCA) was automatically performed through the software (Figure 1). The starting and ending points were manually selected at the proximal segment of the RCA 10–50 mm, LAD, and LCX 40 mm, and the software was fully automated to remove components such as contrast agents, vascular walls, myocardium, and small collateral circulation vessels that were not within the measurement range and segment of the outer wall of blood vessels and the pericoronal fat boundary. Finally, the software’s automatic measurement function was used to calculate the FAI values of perivascular fat in the divided segments (Figure 2). Simultaneously, the same blood vessel was measured three times, and the average value was taken to ensure the reliability and repeatability of the measured data.

### 2.6. Statistical Analysis

According to whether patients experienced adverse coronary events during the follow-up process, they were divided into a less than moderate stenosis non-adverse event group (referred to as the non-adverse event group) and a moderate-to-lower stenosis adverse event group (referred to as the adverse event group). All statistical analyses and results plotting were performed using IBM SPSS (version 20.0). The data are represented as the mean ± standard deviation ($\bar{X}$ ± *S*). The *t*-test was used to compare the differences in peripheral FAI values between the LAD, LCX, and RCA blood vessels in the two groups of patients. When *p* ≤ 0.05, the difference was considered to be statistically significant. For the clinical parameters that had an impact on patients with coronary heart disease, the bias caused by individual factors was corrected using χ^2^ tests and an analysis of covariance to more accurately evaluate the relationship between the coronary FAI and adverse events of coronary heart disease in patients with coronary artery stenosis below the moderate level and the clinical application value of coronary FAI.

## 3. Results

### 3.1. Basic Clinical Information of Patients

A total of 172 patients with moderate or lower coronary artery stenosis were included in this study, including 87 patients in the non-adverse group and 85 patients in the adverse group. The comparison of basic clinical information and biochemical indicators between the two groups is shown in Table 1.

The *t*-test analysis between the two groups revealed that the age difference between the two groups of patients was statistically significant (*F* = 13.89. *p* < 0.001). The group with adverse reactions was older, and there was no statistically significant difference in sex or smoking status between the two groups of patients. Moreover, there was no statistically significant difference in blood biochemical indicators between the two groups of patients (*p* > 0.05).

### 3.2. FAIs of Three Coronary Arteries and Adverse Events of Coronary Arteries

Among the 172 patients, due to image quality factors such as motion artifacts, respiratory artifacts, and stent artifacts, some blood vessels were unable to be recognized by the AI software for 3D reconstruction. A total of 172 LADs, 171 LCXs, and 170 RCAs were ultimately included. The FAI values of the three blood vessels between the two groups of patients were compared using *t*-tests, and the results are shown in Table 2.

The differences in FAI values among the three blood vessels were statistically significant (*p* < 0.05), and the FAI values of the three blood vessels in the group with adverse events were higher than those in the group without adverse events.

Table 1 shows that there was a significant difference in the age of patients between the two groups with or without adverse events, which may have affected the comparison of FAI values of the LAD, LCX, and RCA between the two groups. Therefore, the age of patients is taken as a covariate for analysis. The results showed that after excluding the influence of age, the difference in the FAI values of the LAD, LCX, and RCA between the two groups was still statistically significant (F values were 52.19, 14.90, and 7.60, respectively, *p* < 0.001). The results are shown in Table 3.

## 4. Discussion

### 4.1. Pericardium Fat and Coronary Artery Surrounding Fat

Epicardial fat is a type of pericardial fat that originates from the mesoderm and is supplied by the coronary artery [17,18]. Currently, research shows that epicardial fat has a higher correlation with physiological indicators in cardiovascular disease patients than obesity indicators such as the BMI and waist circumference [19].

Adipose tissue around the coronary artery is a kind of epicardial fat that is close to the myocardium and directly contacts the coronary artery. In the process of coronary atherosclerosis and the occurrence, development, and destabilization of coronary plaque, pericoronary fat plays a role in its regulation [20].

### 4.2. Quantification of Coronary Periarterial Fat and Its Clinical Significance

Currently, traditional imaging-related examinations for coronary heart disease mainly focus on clinical anatomical differentiation. The determination of the degree of coronary artery stenosis is particularly important in the diagnosis and treatment of coronary heart disease, which directly affects the selection of clinical diagnosis and treatment methods [21,22]. CCTA-diagnosed patients with moderate or higher coronary artery and coronary atherosclerotic plaque are obvious, and clinical treatment is more active. In patients with moderate or lower coronary stenosis, atherosclerotic plaque is not obvious, and such patients are generally not treated clinically. In this study, 172 patients were included with suspected coronary heart disease who had been admitted for treatment due to reasons such as chest pain. All of the patients were diagnosed with moderate or lower coronary artery stenosis via CCTA. After follow-up, some patients still experienced adverse coronary events.

A total of 172 patients with or without adverse events were divided into two groups, with 87 patients having no adverse events and 85 patients having adverse events. There were no statistically significant differences in sex composition, smoking, blood sugar, and lipid levels between the two groups. Patients in the group with adverse events were older than those in the group without adverse events. Therefore, commonly used indicators cannot predict the occurrence of adverse events in patients with moderate to lower coronary stenosis. However, after quantifying the patient’s FAI, it was found that there was a significant difference in the FAIs between the two groups of patients; the FAI in the adverse group was greater than that in the non-adverse group. We believe that this is due to changes in the microenvironment of the patients’ coronary arteries, resulting in changes in PCAT.

Traditional research has confirmed that vasculitis has a significant impact on PCAT [23,24,25]. Kohchi et al. [26] also confirmed through dissection of patients who died of coronary heart disease that there are a large number of inflammatory cells in the pericoronal fat of these patients. When inflammation occurs in blood vessels, it inhibits the generation of local fat around adjacent blood vessels, causing changes in the composition of surrounding fat, manifested as a decrease in the density of fat around blood vessels in CCTA images [14]. The phenomenon of an abnormal increase in fat tissue attenuation in CT images caused by edema or inflammatory and/or vegetative infiltration was first described in abdominal and pelvic CT [27]. Based on the results of this study, we believe that the FAI values of patients with adverse events are higher than those without adverse events. This may be due to the early onset of inflammation in the patient’s coronary arteries, which occurs earlier than morphological changes in the patient’s coronary arteries, such as luminal stenosis and plaque formation. Although the coronary lumen of patients is moderately narrow or below, the presence of coronary inflammation can also lead to adverse events during follow-up. Measuring the patient’s coronary FAI can provide new “evidence” for evaluating the patient’s coronary lumen “health” and help predict whether the patient may experience coronary adverse events, which can help clinicians increase their attention to the patient’s health.

Previous studies were mostly limited to the measurement of the local volume and thickness of PCAT due to the reliance on manual processes, which posed challenges in terms of efficiency and consistency. In this study, an innovative approach was employed utilizing algorithms embedded in the software for automatic processing. Specifically, a systematic four-step segmentation process was implemented prior to conducting measurements. These steps involved the detection of the heart and its anatomy, isolation of the heart, extraction of the centerline, and segmentation of the inner and outer vessel wall. Moreover, marginal space learning and steerable feature by Zheng et al. [28] were used for the detection of the heart and its anatomy. Both model— and data-driven approaches by Zheng et al. [29] that took prior information about vessel-specific regions of interest were used for heart isolation. Additionally, robust centerline extraction was facilitated, which was crucial for subsequent analysis. For inner wall segmentation, the algorithm was based on ray-casting and the analysis of a subsequent Markov random field graph with convex priors [30]. For the outer vessel wall, an adaptive self-learning edge model using a combination of 3D and 2D active contour models was employed [31]. It is worth noting that the algorithm mentioned above is currently the best-performing fully automatic approach on the Rotterdam Coronary Challenge leaderboard in their respective areas. These algorithms have undergone rigorous evaluation and have been proven to be efficient and accurate in their performance.

The results of this study showed that there were no significant differences in sex, smoking status, or many biochemical indicators between the two groups of patients. Only the age and the FAI of the patients showed significant differences; after adjusting for bias with the age of the patients as a covariate, the FAIs between the two groups of patients still showed significant differences. Moreover, the FAI of the patient group with adverse events was significantly higher than that of the group without adverse events, which may be due to the inhibition of the composition and content of perivascular fat by inflammatory factors around the coronary artery, resulting in changes in uptake and metabolic function [32,33]. These results are consistent with those of Hirata et al. [34], who found that the severity of coronary heart disease is correlated with the type and proportion of inflammatory cells in PCAT. However, there are differences between the two research methods, as shown in Table 4. Our research utilized machine learning to provide a simple, repeatable, and non-invasive approach to the overall changes in coronary artery FAI. These results can accurately reflect the overall changes in the patient’s coronary vascular status, and the operator requirements are lower. This machine learning-based approach has better prospects for popularization and utilization in clinical research.

The results of this study show that the FAI is a parameter that can more accurately reflect the vascular condition of patients in CAD compared to traditional high-risk factors such as BMI, age, sex, etc. The measurement of FAI enables clinicians to provide more reference information on patients’ coronary artery health in 2D (such as the extent of lumen stenosis, wall atherosclerosis, etc.). When the patient’s FAI increases, it indicates that they may have coronary inflammation. To prevent adverse coronary events from occurring, it is important to pay more attention to the patient and to follow up frequently, even if the degree of coronary stenosis is only mild or even non-existent. In previous coronary CCTA examinations, the subjective influence of diagnostic physicians was significant. However, using machine learning to measure FAI not only simplifies the operation and has high repeatability, but also significantly reduces the subjective judgment of operators, which greatly helps to improve the consistency of imaging reports.

### 4.3. The Novelty and Limitations of This Study

This study is a retrospective study with a large sample size and a long follow-up time, and the use of machine learning is a characteristic of this study. The parameter observed in this study is FAI, which is a relatively novel biological indicator that differs from the volume, thickness, and density of PCAT observed by previous researchers. Quantifying the coronary FAI in CCTA images through machine learning algorithms is simpler and more accurate than traditional manual delineation methods used by domestic and foreign researchers. The measurement results also reflect the FAI value of the entire coronary artery. This novel AI-based examination method has good repeatability, the results are more objective and less time-consuming, and it has good clinical application prospects.

This study has certain limitations: ① this was a retrospective study on imaging images, which does not have reproducibility for the source of patients; and ② due to resource constraints, there may have been patient classification bias during the follow-up process, leading to statistical bias. This was a single-center study, with most of the research subjects being Han Chinese from the southwest region. The next step will be to continue exploring these issues in subsequent research.

## 5. Conclusions

As the most widely used imaging examination method in clinical practice, CCTA only reflects changes in coronary artery morphology in the current imaging diagnosis approach for coronary heart disease. This study used AI software to evaluate the FAIs of patients with moderate to low coronary artery stenosis. There was a significant difference in FAI between patients who experienced coronary adverse events and those who did not. Therefore, we believe that the measurement of FAI can reflect the phenotype and metabolic activity of PCAT to a certain extent, indirectly revealing changes in the microenvironment around the coronary artery to provide more reference imaging evidence for the prevention and treatment of coronary heart disease.

## Figures and Tables

**Table 1:** Comparison of basic clinical data and biochemical indicators between the two groups of CAD patients.

Variable	Without Adverse (*n* = 87)	With Adverse (*n* = 85)
Basic clinical data		
Age (years)	61.26 ± 9.41	65.01 ± 12.43
Sex (male:female)	47:40	47:38
Smoking history (Yes:No)	31:56	31:54
Biochemical indicators		
Fasting blood glucose (mmol/L)	5.80 ± 1.74	5.66 ± 1.41
Total cholesterol (mmol/L)	4.63 ± 1.07	4.13 ± 1.05
Low-density cholesterol (mmol/L)	2.63 ± 0.83	2.42 ± 0.87
High-density cholesterol (mmol/L)	1.41 ± 0.45	1.33 ± 0.39
Triglycerides (mmol/L)	1.76 ± 1.66	1.39 ± 0.88

Numerical variables are expressed as the average ± standard deviation.

**Table 2:** Comparison of FAI values of three coronary arteries between the two groups of patients.

	Without Adverse			With Adverse			*t*	*p*
	*n*	$\bar{X}$	S	*n*	$\bar{X}$	S		
LAD	87	−44.95	4.10	85	−39.36	5.89	−7.212	<0.001
LCX	86	−36.52	4.11	84	−33.72	5.32	−3.844	<0.001
RCA	86	−44.71	6.61	84	−41.70	7.59	−2.752	0.022

**Table 3:** Comparison of FAI values after adjusting for age differences between the two groups of patients.

	Without Adverse			With Adverse			*F*	*p*
	*n*	$\bar{X}$	S	*n*	$\bar{X}$	S		
LAD	88	−44.99	4.18	85	−39.38	5.93	52.19	<0.001
LCX	88	−36.44	4.12	84	−33.76	5.42	14.90	<0.001
RCA	87	−44.70	6.63	84	−41.91	7.50	7.60	<0.001

**Table 4:** Different comparisons between two groups of studies.

	Zhang et al.	Hirata et al. [34]
Study measurements	Coronary artery FAI	EAT was taken from the anterior wall of the left ventricle
Study method	Machine learning	Surgical intervention
Traumatic examination methods	No	Yes
Ease of operation	Easy	Difficult

## Data Availability

We do not share our data.
